# From a meso- to micro-scale connectome: array tomography and mGRASP

**DOI:** 10.3389/fnana.2015.00078

**Published:** 2015-06-04

**Authors:** Jong-Cheol Rah, Linqing Feng, Shaul Druckmann, Hojin Lee, Jinhyun Kim

**Affiliations:** ^1^Korea Brain Research InstituteDaegu, South Korea; ^2^Department of Brain Science, Daegu Gyeongbuk Institute of Science and Technology (DGIST)Daegu, South Korea; ^3^Center for Functional Connectomics, Korea Institute of Science and Technology (KIST)Seoul, South Korea; ^4^Janelia Farm Research Campus, Howard Hugh Medical InstituteAshburn, VA, USA; ^5^Neuroscience Program, University of Science and TechnologyDaejeon, South Korea

**Keywords:** connectome, mGRASP, array tomography, 3D atlasing, multiple scales

## Abstract

Mapping mammalian synaptic connectivity has long been an important goal of neuroscience because knowing how neurons and brain areas are connected underpins an understanding of brain function. Meeting this goal requires advanced techniques with single synapse resolution and large-scale capacity, especially at multiple scales tethering the meso- and micro-scale connectome. Among several advanced LM-based connectome technologies, Array Tomography (AT) and mammalian GFP-Reconstitution Across Synaptic Partners (mGRASP) can provide relatively high-throughput mapping synaptic connectivity at multiple scales. AT- and mGRASP-assisted circuit mapping (ATing and mGRASPing), combined with techniques such as retrograde virus, brain clearing techniques, and activity indicators will help unlock the secrets of complex neural circuits. Here, we discuss these useful new tools to enable mapping of brain circuits at multiple scales, some functional implications of spatial synaptic distribution, and future challenges and directions of these endeavors.

## Introduction

It is no exaggeration to state that the beautiful drawings of the visionary Spanish neuroanatomist Santiago Ramon Cajal (1852–1934, Nobel Laureate 1906) illustrating neuronal structure and brain architecture, set the standard for neuroanatomy in the last century. Primary insights emerging from this and subsequent work are that the brain is a network of diverse types of neurons and glial cells communicating with one another mainly through synaptic connections, and that anatomical connections provide the structural framework for information processing in the brain. Comprehensive knowledge of the brain’s wiring in complex neuronal circuits at both meso- (region-by-region) and micro-scales (synapse-by-synapse) is essential for understanding brain functions. In an era of advanced modern technologies including ever-increasing computer power, neuroanatomy for the XX1st century, aims to determine the complete connectomes (neural wiring diagrams) of several key species (i.e., human, mouse, fruit fly, worm, etc.; Chklovskii et al., 2004; Sporns et al., 2005; Behrens and Sporns, 2012; Oh et al., 2014). Recently, there has been much excitement about new techniques for establishing the brain-wide, cellular-level, meso-scale connectome for the mouse using injections of fluorescent protein-expressing virus and tracers (Hunnicutt et al., 2014; Oh et al., 2014; Pollak Dorocic et al., 2014; Zingg et al., 2014). These systematic and standardized approaches allow spatial registration of mesoscopic connectivity data from separate experiments into a collective 3D reference space, while computational analyses of connection strength in 3D topography provides a whole-brain connectivity matrix. These meso-scale connectome atlases of brain-wide tractography of defined cell-types in defined regions are freely available, providing a foundational resource for structural and functional investigations into neural circuits that underlie complex brain functions, such as behavioral and cognitive processes. To solve big puzzles of the brain, however, the meso-scale connectome is insufficient and micro-scale synaptic connectivity remains substantially unknown.

It is a challenging, ongoing task to map synaptic connectivity in the brain, a complex and compact tissue composed of thin (<1 μm in diameter) yet long (often >1 mm in length) neuronal processes from densely packed neurons, communicating with each other through synapses on the scale of nanometers (~20 nm). Mapping these structures requires advanced neuronal labeling, imaging, and reconstructing techniques that provide high-resolution in multiple scales (Box 1; Kleinfeld et al., 2011; Wickersham and Feinberg, 2012; Morgan and Lichtman, 2013; Yook et al., 2013). Traditional methods based on electron microscopy (EM) that offer high resolution on the nanometer scale have been used to find and characterize synapses, but these approaches lack the throughput capacity to reconstruct even a small portion of the connection matrix. Thus, despite recent advances, these approaches remain practical only for very small volumes (Knott et al., 2008; Bock et al., 2011; Briggman et al., 2011; Kreshuk et al., 2011). Recently, to circumvent time- and labor-intensive EM-based approaches and the low resolution of light microscopy (LM), researchers have developed fluorescence-based approaches combined with sophisticated genetic and optical methods (Livet et al., 2007; Micheva and Smith, 2007; Wickersham et al., 2007; Micheva et al., 2010; Cai et al., 2013; Yook et al., 2013). Of several advanced LM-based connectome technologies, array tomography (AT) and mammalian GFP-Reconstitution Across Synaptic Partners (mGRASP) can provide relatively high-throughput mapping synaptic connectivity at multiple scales. AT combines LM and EM approaches to resolve synapses by using multiple antibodies to label synaptic markers (Micheva and Smith, 2007; Micheva et al., 2010). It benefits from the high throughput of LM, high *z*-resolution of EM, and improved quantitative reliability of information obtained through multi-immunofluorescence. Furthermore, repeated cycles of antibody stripping and re-staining synaptic components provides a single-synapse analysis, or a synaptogram, that offers insights into synapse molecular diversity (Micheva et al., 2010). Meanwhile, mGRASP (Kim et al., 2011; Feng et al., 2012), synapse-specific labeling with two complementary GFP components, provides suitable tools for mapping mammalian synaptic connectivity at multiple scales: micro-scale for synapse-by-synapse or neuron-by-neuron measures, and meso-scale for local and long-range neuronal projections mappings. In addition, new online resources^1^ (Oh et al., 2014; Zingg et al., 2014) provide useful references with brain-wide coverage and guidance for further detailed circuit mapping at the micro-scale, which can be accomplished by AT, and mGRASP-assisted circuit mapping (ATing and mGRASPing, respectively) which we review below. Most recently, using these two methods, we generated a comprehensive fine-scale circuit mapping of hippocampal and somatosensory cortical regions showing new spatially-structured synaptic connectivity patterns (Rah et al., 2013; Druckmann et al., 2014). Increasingly, studies reveal that nonrandom organization of interconnectivity exists to some degree within the nervous system at multiple scales, including individual neurons, groups of neurons, architectonic regions, and functional systems (DeBello et al., 2014).

Box 1 **Brain mapping methods.**

We summarize the currently available methods to enable mapping of brain circuits at the scale levels (from macro- to micro-scale): (1) Non-invasive brain imaging methods, i.e., MRI and PET widely used for the whole human brain, provide information about region-to-region connectivity at millimeter-resolution (Catani and Thiebaut de Schotten, 2008). (2) High-throughput serial block face two-photon tomography (Ragan et al., 2012) has been used to achieve the meso-scale connectome of the whole mouse brain (Oh et al., 2014). (3) Conventional EM imaging has been considered as a gold-standard method to resolve the nanometer-scale synapses yet its low-throughput and reconstruction difficulty constrain achievable brain volume. (4) Therefore, much effort has been devoted to improving the EM-based imaging approach. Large-scale EM imaging techniques (e.g., Serial Block Face Scanning EM, Automated Tape-Collecting Lathe Ultra Microtome, Camera array, Focused Ion Beam Scanning EM) have been recently developed and demonstrated to enable dense reconstruction of local circuit (Merchán-Pérez et al., 2009, 2014; Bock et al., 2011; Briggman et al., 2011; Briggman and Bock, 2012; Blazquez-Llorca et al., 2013; Hayworth et al., 2014). Although these methods, at present, remain relatively time-consuming and volume limited, further advances in these techniques may allow for complete connectome in the large-scale (Hayworth et al., 2015). (5) For functional assessment along with connectivity, laser-scanning photostimulation (e.g., glutamate uncaging and optogenetic approach) combined with electrophysiological recordings have accelerated mapping region-to-cell and cell-to-cell connectivity together with measures of synaptic efficacy and strength (Shepherd and Svoboda, 2005; Shepherd et al., 2005; Yoshimura and Callaway, 2005; Yoshimura et al., 2005; Petreanu et al., 2007, 2009; Ashby and Isaac, 2011; Fino and Yuste, 2011; Hooks et al., 2011; Mao et al., 2011). A potential concern, though, is that these methods can yield ambiguous results owing to the low precision of uncaging and the low resolution of photostimulation. (6) Advanced LM-based circuit mapping tools such as AT and mGRASP can provide relatively high-throughput mapping synaptic connectivity at multiple scales. Details of these two methods are described in this review.

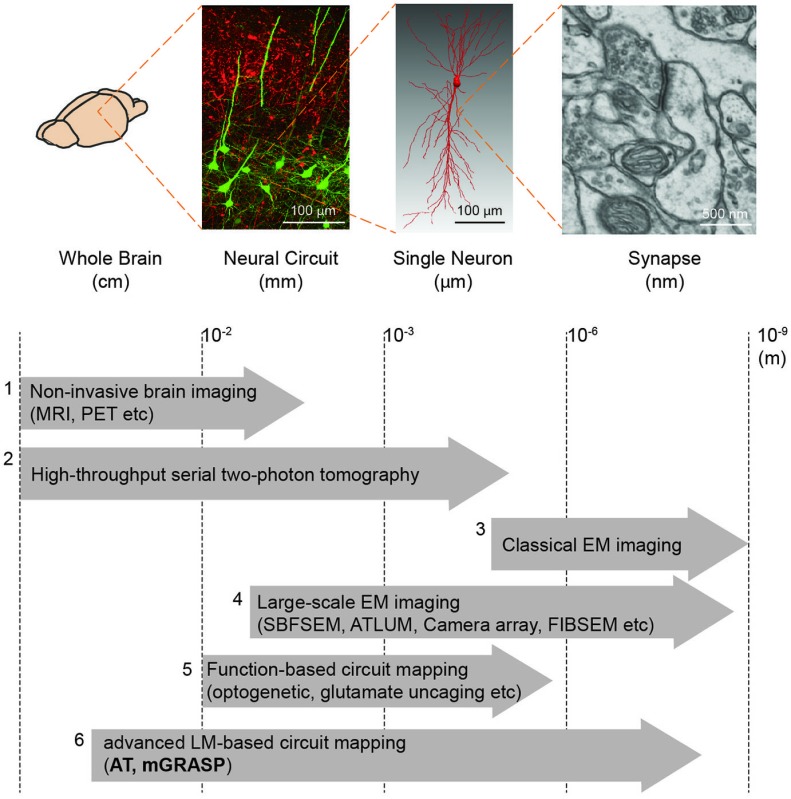

The recent development of powerful tools for relatively high-throughput mapping of synaptic networks promises major advances in understanding brain functions. Yet, mapping neuroanatomical connectivity in most model organisms remains difficult owing to technical challenges and gaps between connectome mapping enterprises. Creating ties between meso- and micro-scale maps and turning anatomical connectivity maps to comprehensive knowledge remain difficult. Here we discuss useful new tools to enable mapping of brain circuits at multiple scales, some functional implications of spatial synaptic distribution, and future challenges.

### Array Tomography-Assisted Circuit Mapping (ATing)

**Figure 1:**
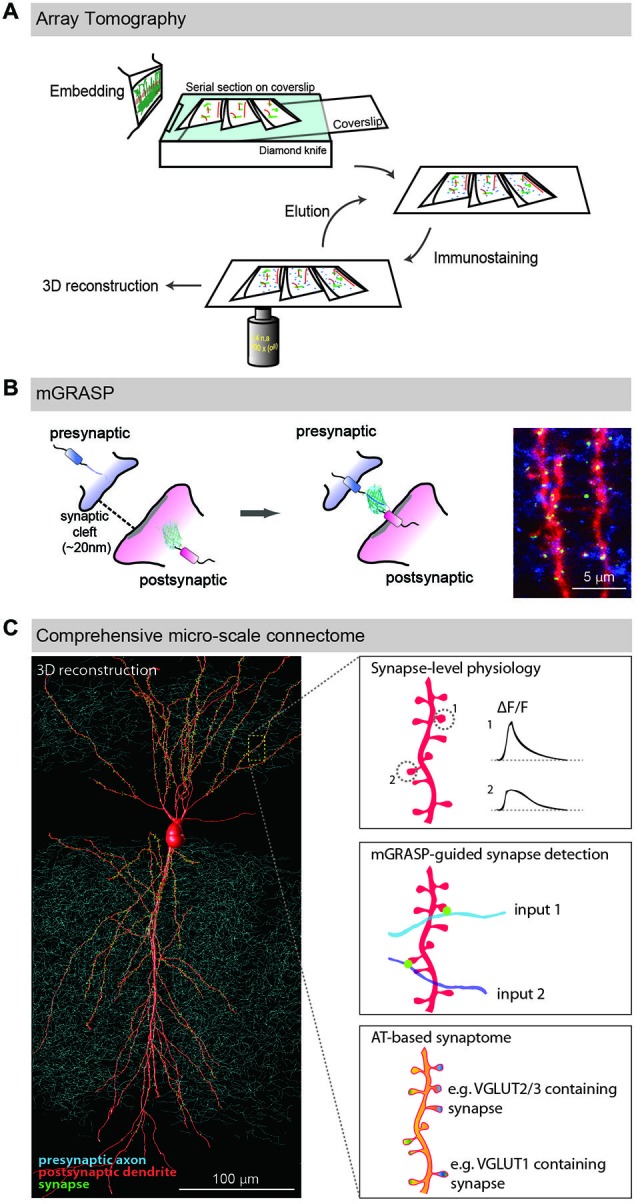
**LM-based circuit mapping. (A)** Schematic illustration of Array tomography. Embedded brain tissue is serially sectioned into nanometer ultrathin slice array and repeatedly immunostained with multiplex synaptic makers. Then the stained serial sections are imaged and computationally reconstructed into isotropic three-dimensional structure. **(B)** Schematic illustration of mGRASP. Two complementary split-GFP fragments, non-fluorescent are tethered to each side of synaptic membranes. When the two neurons form synapses, thus closely opposed across a synaptic cleft, fluorescent GFP is reconstituted. Example image shows mGRASP signals (Green) in hippocampal CA3 (blue) -to-CA1 (red) synapses. **(C)** Schematic illustration of comprehensive micro-scale connectome with functional assessments. Physiological characteristics of individual synapses accessed by two-photon microscopy and electrophysiology, presynaptic input sources by mGRASP or anterograde labeling in combination with AT-based synaptogram will reveal relationships of the proteomic diversity, function and input sources at the level of individual synapses. Eventually one will be able to estimate functions and input sources of synapses based on AT images. (Panels **A,B** are modifications of the figures from Kim et al., 2011; Rah et al., 2013, respectively).

AT was developed to image synaptic architecture and neuronal circuits (Micheva and Smith, 2007; Micheva et al., 2010; Rah et al., 2013). It is achieved by repeated wide-field immunofluorescence imaging of arrays of ultrathin serial brain sections, followed by computational reconstruction into an isotropic three-dimensional (3D) volume (Figure 1A). Although optical sectioning by confocal or two-photon microscopy allows imaging of thick brain sections and reconstructing neuronal structures from the obtained images, its resolving power is inadequate to resolve nanometer-scale synapses, mainly because it provides poor *z*-resolution. As this technique uses pixel-thick or thinner brain sections (50~200 nm), the *z*-axial resolution is determined by not Abbe’s rule but the thickness of the sections. Another significant advantage of ultrathin physical sectioning is that it circumvents some technical hurdles including the laser and antibody penetration problems that often limit the usefulness of immunofluorescence staining and imaging in thick brains sections. AT enables detailed and reliable investigations of the proteomic diversity of individual synapses by using repeated cycles of antibody stripping and re-staining with a large number of multiplex of synaptic makers, resulting in a comprehensive description called a synaptogram (Micheva et al., 2010).

AT reveals not only anatomical circuits but also the synaptic proteome, thus offering insights into synaptic physiology. It has been suggested that synapse-level physiology can be inferred from molecular information of individual synapses (Yasuda et al., 2006). A good example of this approach is the physiological relevance of the expression patterns of vesicular glutamate transporter isoforms, i.e., VGLUT1 and 2 (Wojcik et al., 2004; Moechars et al., 2006; Fioravante and Regehr, 2011). Intensive anatomical analysis with LM and EM showed that the expression of these two major isoforms of VGLUT is spatially and temporally distinctive (Boulland et al., 2004; Graziano et al., 2008; Lei et al., 2013). Interestingly, the expression levels of VGLUT1 and VGLUT2 affect specific forms of signal processing like short-term plasticity by controlling quantal size and neurotransmitter release probability (Wojcik et al., 2004; Moechars et al., 2006; Fioravante and Regehr, 2011). These findings raised perplexing questions since other studies demonstrated that these isoforms are equally effective in transporting glutamate into vesicles (Fremeau et al., 2004). This puzzle was solved by identifying an additional synaptic molecule, endophilin A1, and determining its distinct interplay with VGLUTs (Weston et al., 2011). Endophilin A1, a positive regulator of glutamate release, is inhibited by VGLUT1 but not VGLUT2 or 3 so that synapses with VGLUT2/3 have higher vesicular release probabilities. Thus, the quantitative synapse proteome together with circuit maps using AT will help provide synapse-level physiological information including type of inputs, synaptic strength, and plasticity.

A potential drawback of AT, though, is its limited accuracy of synapse detection (Micheva et al., 2010; Rah et al., 2013). This occurs because the lateral resolution of AT is still agonized by the light diffraction limit while the *z*-axial resolution of AT is determined solely by the section thickness, which is as good as that of conventional TEM. The accuracy AT reaches for synapse detection has been estimated and validated in thalamocortical tissue by a synapse-by-synapse comparison with results obtained by EM, taking advantage of the ready comparability of AT to EM: Up to ~80% of thalamocortical synapses detected by AT were validated by TEM and 86% of total TEM-identified synapses were detected by AT (Rah et al., 2013). Although the current level of accuracy of synapse detection by AT is considerably greater than that of traditional LM-based approaches (approximately 20–50%), we expect that AT accuracy can be improved by combining it with advanced optical methods or/and supplementary synapse labeling methods. Given the success of recent advanced super-resolution LM techniques such as PALM (Betzig et al., 2006), STORM (Rust et al., 2006), SIM (Gustafsson, 2005), and STED microscopy (Willig et al., 2006) allowing multi-fluorescence imaging with nanometer resolution, there are now increasing efforts to combine super-resolution microscopy with AT. In fact, it has been demonstrated that STORM and STED are compatible with AT to enhance lateral resolution up to the single molecular level (Punge et al., 2008; Nanguneri et al., 2012). Another way to improve the accuracy of synapse detection by AT, possibly together with high-resolution imaging too, is to make use of additional and improved synapse labeling strategies instead of using only immunostaining with synaptic markers. The main reasons AT achieves relatively low accuracy in detecting synapses are the high density of synapses in the brain (~1/μm^3^; Schüz and Palm, 1989; DeFelipe et al., 1999) and the relatively low antibody specificity for synaptic vesicle proteins. A theoretical study predicted that the labeling of synapse specific proteins (e.g., PSD-95, piccolo, bassoon), instead of synaptic vesicle proteins could detect 95–99% of all synapses (Mishchenko, 2010). Recently developed stoichiometric endogenous labeling of synaptic proteins, called endogenous labeling via exon duplication (ENABLED), may provide a promising way to solve these problems (Fortin et al., 2014). Thus, when combined with other advanced optical and supplementary synapse labeling methods, ATing offers unique fundamental synaptic molecule profiles that may be used to describe neuronal networks. We will further discuss the marriage of AT and mGRASP in the following sections.

### mGRASP-Assisted Circuit Mapping (mGRASPing)

mGRASP is a genetically-controlled, molecular engineering method to detect mammalian synapses using LM (Kim et al., 2011; Feng et al., 2012, 2014; Druckmann et al., 2014). It is based on two complementary split-GFP fragments (called spGFP1-10 and spGFP11), separately non-fluorescent, each tethered to synaptic membranes in each of two neuronal populations. When two neurons, each expressing one of the fragments, are closely opposed across a synaptic cleft, the split fragments unite and fluorescent GFP is reconstituted in that location (Figure 1B). This molecular engineering approach allows the resolution, at nanometer-scale, of synapses viewable by LM.

The GRASP technique was initially implemented in *C. elegans* (Feinberg et al., 2008). Recently, we successfully adapted mGRASP for the more complex synapses of mammals by optimizing the synaptic transmembrane carriers (Kim et al., 2011). We achieved this by engineering spGFP carriers that are specifically targeted to synaptic membranes, and that accommodate the physical spacing of the synaptic cleft to precisely label actual synapses, not non-synaptic membrane contacts. The manifest benefit of mGRASP technology is that it can rapidly and accurately detect nanometer-scale (~20 nm) synapses despite the diffraction limitations of LM: using this technique, fluorescence indicates the locations of mammalian synapses quickly, confidently, and with high spatial resolution. When tested with known synaptic and non-synaptic connections in samples full of axonal contacts, mGRASP was shown to specifically detect actual synapses with very few false positives. When combined with specialized analysis software (Feng et al., 2012, 2014, 2015), mGRASP can relatively quickly reveal the precise locations and numbers of synapses along postsynaptic dendrites, sites responsible for determining many important characteristics of signal processing.

More recently, using our mGRASP technology, we performed a comprehensive fine-scale circuit mapping of hippocampal regions and identified new patterns of spatially-structured synaptic connectivity (Druckmann et al., 2014). An advantage of mGRASP technology is that, when used with an improved computational analysis, it can map mammalian synaptic connectivity at multiple scales: micro-scale for synapse-by-synapse or neuron-by-neuron measures, and meso-scale for local and long-range neuronal projection measures. A potential concern, though, is that this technique sometimes registers false negatives, making it difficult to determine absolute numbers of synapses. The problem of false negatives is common to all LM approaches and varies with instrumental parameters (e.g., laser power, emission spectra, etc.). Further optimizing of mGRASP technology and applying it in combination with other technologies will lead to useful new tools for mapping mammalian synaptic connectivity. We recently offered a step-by-step protocol for mGRASP to map synaptic connectivity in the mouse brain (Feng et al., 2014). Although our technique uses combinations of well-established experimental methods (e.g., virus production, *in utero* electroporation, stereotaxic injection, brain slice preparation, and confocal imaging), in practice, each experimental step needs to be adjusted specifically to make mGRASPing effective. Our well-optimized protocol allows the rapid and precise characterization of synaptic connectivity in neuronal circuits of both healthy and pathological tissues, potentially aiding in the diagnosis of abnormal synaptic connectivity.

Furthermore, creative combinations of mGRASP with currently available techniques for imaging mammalian synaptic connectivity will contribute substantially to brain mapping, since thus far, none of these currently available techniques for imaging mammalian synaptic connectivity is in itself perfect (Yook et al., 2013). The stochastic multicolor labeling of Brainbow combined with mGRASP, for instance, could identify the presynaptic partners of a given neuron; it would require labeling each neuron and preparing dense-reconstructions of synaptic connectivity under LM (Cai et al., 2013). Together with new optical clearing methods (Chung and Deisseroth, 2013; Ke et al., 2013; Renier et al., 2014; Susaki et al., 2014) or the very recently developed expansion imaging method which uses the physical expansion (~4.5-fold) of tissue, resulting in physical magnification (Chen et al., 2015), mGRASPing with multicolored axonal labeling allows mapping connectivity from multiple inputs. Also, mGRASP combined with a new retrograde label virus system (Kato et al., 2011a,b) could help unlock the secrets of disynaptic circuits as well as monosynaptic pairs of cells. Further, a common drawback of all methods for anatomical synaptic mapping, the lack of information about synaptic activity and strength, can be overcome through combinations of techniques including existing activity indicators and optogenetic tools.

### Comprehensive Micro-Scale Connectome with Triple Combination of AT, mGRASP, and Activity Sensors

Given the distinctive advantages and pitfalls of both AT and mGRASP, we propose the combination of these two technologies into a powerful tool for determining the micro-scale connective synaptome. As described above, ATing is beneficial for revealing detailed synapse proteomes but is hampered by limited accuracy of synapse detection compared to those achievable through more laborious procedures. Meanwhile, mGRASPing provides high-throughput and accurate synapse detection but provides no information about synaptic molecular diversity. A marriage of these two complementary technologies would provide powerful descriptions of neuronal circuits. In principle, advanced mGRASP can accurately and rapidly detect specific synapses in a particular connection and AT can subsequently denote molecular profiles of the synapses. This approach will not only enhance the accuracy of ATing but also provide fundamental information of complex neuronal networks for understanding brain functions.

It is widely believed that the number, morphology, and molecular compositions of synapses intimately related to synaptic functions. And, abnormalities in synaptic number, shape, and compositions have been demonstrated to be accompanied by synaptic dysfunctions in many neurological disorders such as Alzheimer’s disease (Selkoe, 2002), Parkinson’s disease (Calabresi et al., 2006), Schizophrenia (Stephan et al., 2006), Fragile X syndrome (Pfeiffer and Huber, 2009), and Rett syndrome (Chao et al., 2007). Also, growing lines of evidence show that a functional balance of excitatory and inhibitory systems is fundamental for the healthy function of brains by providing for the fine tuning of neuronal circuits (Wehr and Zador, 2003; Xue et al., 2014); disruption of excitatory-inhibitory (E-I) balance engenders neurological disorders such as autism spectrum disorder, schizophrenia, and epilepsy (Chao et al., 2007; Kehrer et al., 2008; Dudek, 2009). Therefore, it is important to precisely map the number, distribution, and molecular profiles of excitatory and inhibitory (and possibly modulatory as well) synapses in healthy and pathological brains. A combination of AT and mGRASP is suitable for this task.

All of the currently available techniques for imaging mammalian synaptic connectivity provide fundamental structural descriptions but none provide direct assessments of function, such as synaptic strength and efficacy (Yook et al., 2013). A long-term goal in neuroscience is to understand how neuronal activities convey information through network connections. To understand the relationship between the structure and function of neuronal networks, recent studies have attempted to combine EM-based reconstruction with calcium imaging or simultaneous multiple whole cell recordings that can detect active synapses (Bock et al., 2011; Briggman et al., 2011; Ko et al., 2014). However, the issue of relatively low throughput including reconstruction and difficulty in finding cell-to-cell or branch-to-branch correspondence between functional images and reconstructed structural images hinders functional connectivity mapping in large volumes. High-accuracy ATing guided by mGRASPing could serve this purpose and help generate the functional connectome along with synaptome. We propose, for example, high-accuracy ATing guided by mGRASPing in barrel cortex. Barrel cortex is a well-studied multisensory integration system conveying information from whisker movements and object touches into an organized laminar architecture. These features will allow us to study relationships between the structure and function of a network associated with known behaviors (Larkum et al., 2004; Hill et al., 2011) by use of multi-colored calcium sensors (Akerboom et al., 2013; Oheim et al., 2014), followed by high accuracy AT-based circuit reconstruction (i.e., mGRASP aided AT). Recently, it has been shown that accurate subcellular synaptic distributions can be reconstructed on a similar scale using AT and mGRASP in vS1 and hippocampus, respectively (Rah et al., 2013; Druckmann et al., 2014).

### Comprehensive 3D Whole-Brain Atlas at Multiple Scales

Thus far, we have described imaging methods useful for mapping synaptic connectivity and their applications for deciphering functional features of the structural map. The connectome project at multiple scales will necessitate further development of algorithms to reliably extract wiring information from digitized images, and to bring data from different sections and animals into register with one another. Furthermore, creating ties between meso-scale and micro-scale datasets is essential. To achieve this, three main steps are required: (1) Photomicrographs from an individual animal must be registered in 3D while accounting for tissue distortions. (2) Labeled axonal segments must be detected to determine the meso-scale connectome. This step is somewhat challenging, and typically relies on manual or software-assisted tracing, although progress has been made toward providing automated, quantitative estimates of axonal length and density. (3) Individual neurons and their full distributions of synapses must be represented in a common framework to determine the micro-scale connectome. At present, the largest gap between meso- and micro-scale connectome datasets is the difference in data representation used for each. Micro-scale connectome data consist of individual reconstructed neurons and their synapses, while meso-scale connectome data usually consist of the densities of axonal projections from one injection site to another, typically summarized by connectivity matrix as a list of projection tables. The rows and columns of these tables are grouped brain regions, and the table’s data represent the strength of connections between segmented brain regions which can be quantified by the intensity of labeled axons either subjectively by manual rating (Hunnicutt et al., 2014; Zingg et al., 2014), or objectively by image segmentation (Oh et al., 2014; Pollak Dorocic et al., 2014). To reconcile meso- and micro-scale data into a hierarchical structure, all processed data must be mapped to a common 3D reference space (such as the Allen Institute mouse reference atlas).

**Figure 2:**
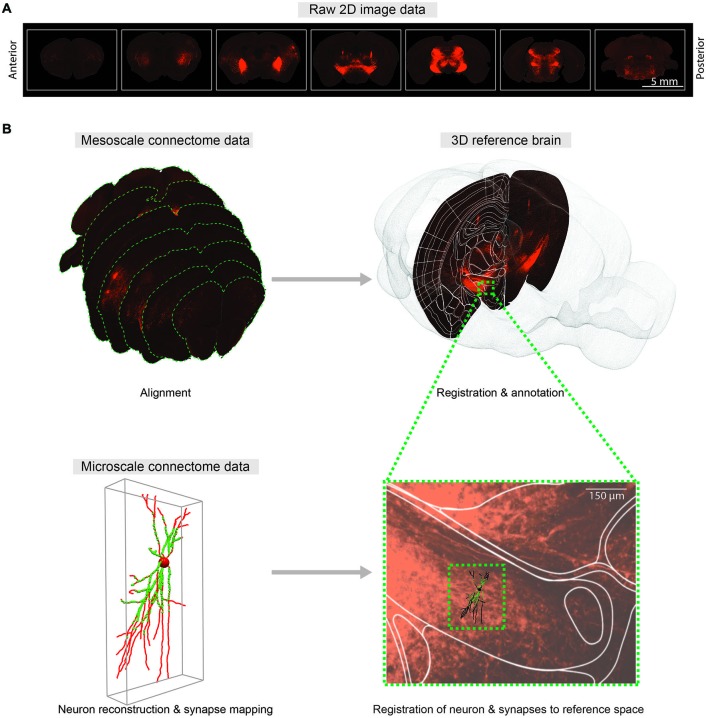
**Comprehensive 3D whole-brain atlas at multiple scales. (A)** Representative coronal sections of the mouse brain with 400 μm intervals showing fluorescent axonal projections for meso-scale connectome. **(B)** Mapping meso- (*top*) and micro-scale (*bottom*) data into a common 3D reference atlas pace. Meso-scale sections are aligned and registered to the Allen Institute Mouse Reference Atlas Space (P56). At micro-scale level, neuTube-reconstructed individual neuron and synapses (Feng et al., 2015) are mapped to the same reference atlas space.

Integrating connectivity information into a reference space requires registration or co-registration of raw brain slice images to the reference brain images, which in turn provide anatomical annotations of raw brain images. In practice, manual annotation seems to provide the most accurate anatomical information (Zingg et al., 2014), yet better automatic algorithms will be required as data sets grow. The purpose of registration is to find the one-to-one mapping or transformation between pixels of brain slice images and pixels of the reference brain images. Main components of the registration framework are referred to as transform, metric and optimizer: transform defines the parameterization of pixel mapping; metric measures the quality of transformations; and optimizer drives the parameters of transformation to reach the best possible alignment by seeking optimal metric values. As the deformations of brain structures between animals are highly variable, non-rigid transformations are required to model the pixel mapping (Figure 2).

B-spline transformation has emerged as a popular choice for modeling non-rigid transformation. A uniform grid of control points forms the local support of the B-spline pixel transformation, and the transformation of each pixel can be calculated from only a few of its neighboring control points. These advantages make B-spline transformation suitable for modeling the local deformations of brain structures. In fact, B-spline based non-rigid registration with mutual information and smooth constraints as metric has been applied in several previous studies of mesoscopic connectivity mapping (Oh et al., 2014; Pollak Dorocic et al., 2014).

Because brain slice images and reference brain images are usually acquired using different methods and protocols, mutual information that can measure the similarity between images of different modalities is useful as a metric. Smooth constraints are used to avoid irregular transformations on homogenous parts of images. Many factors can influence the registration result, including spacing between grid points, intensity levels for mutual information, balance between mutual information and smooth constraints, choice of optimization algorithm, etc. A carefully designed registration strategy may include parameter tuning, coarse-to-fine, global-to-local or intermediate registration targets. This registration framework works well with large brain structures that have distinct intensity levels and that have been validated by checking the location deviation of several brain landmarks such as Area Postrema, Medial Mammillary Nucleus, and Arbor Vitae (Oh et al., 2014). However, it must be evaluated on a case-by-case basis whether the registration framework accurately delineates small neighboring brain regions with similar intensity levels. As there is no other information to guide the registration in such regions, the registration results must be guided mostly by manually tuned smooth constraints rather than the image signals. Quantitative evaluation like Klein et al. (2009) and Ou et al. (2014) is essential for choosing an appropriate registration method and setting optimal parameters.

To merge micro-scale connectome data using mGRASP or AT with meso-scale data, reconstructed neurons and their synapses should also be mapped to a reference space (Figure 2B). Intermediate meso-scale images can be utilized as guidance to locate neurons in the reference 3D space. Once correctly mapped, these micro-scale data can be indexed and efficiently queried by space-partitioning data structures. This pipeline may allow us to merge connectome data on different scales from different research groups into unified hierarchical anatomical structures. We believe that comprehensive cross-referencing of connectivity data from different scales into the same reference space will allow us to explore intermingled neuronal networks at multiple scales and will facilitate understanding of circuit functions.

### From Neuroanatomical Connectivity Maps to Neuro-Knowledge

Understanding the dynamics of neuronal circuits is crucial for studying information processing by these circuits. Knowledge of neuroanatomical connectivity in a comprehensive 3D brain atlas (i.e., topographic axonal projections on the meso-scale and spatial synaptic distributions on the micro-scale) will help in the extraction of dynamics of neuronal circuits. Parceling out brain regions according to their connectivity can serve to define neural circuits, situated between the level of the single neuron and that of the entire circuit.

**Figure 3:**
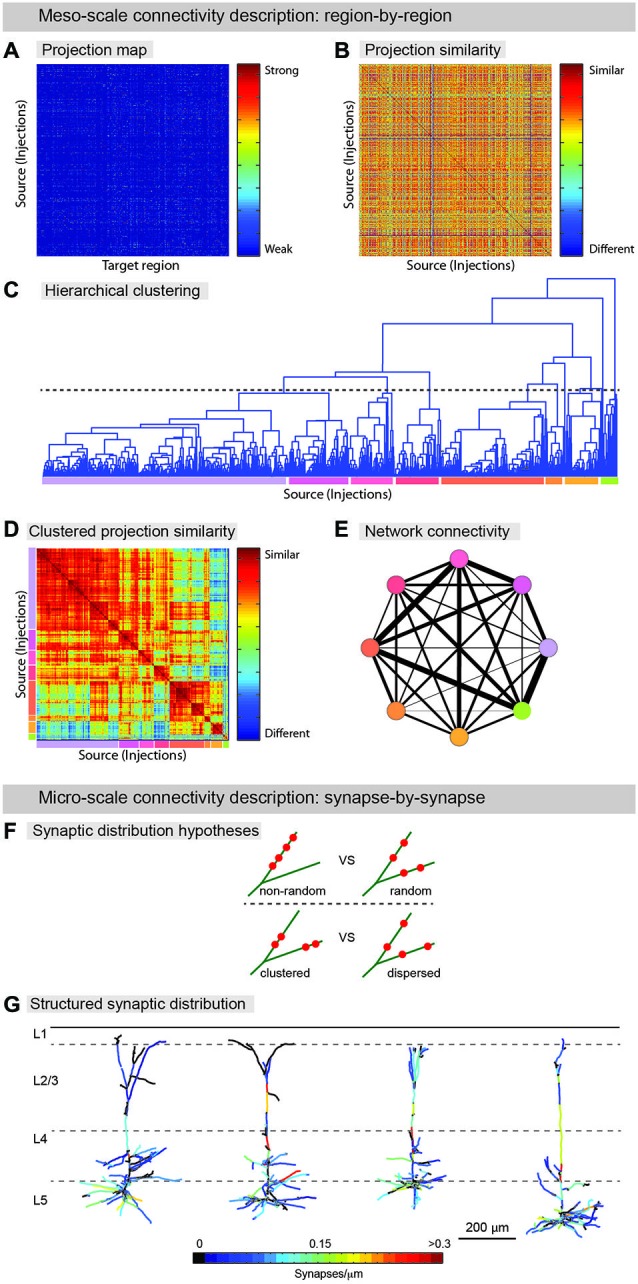
**From neuroanatomical connectivity maps to neuro-knowledge. (A–E) Computational analysis of meso-scale data yields coarse grain network description. (A)** Data from registered meso-scale image data (see Figure 2) is expressed as a map of projection intensity from source to target regions. Each row shows the projection signals from each injected brain to target brain regions based on an atlas. Projection strength is indicated by colors. **(B)** Projection map transformed to show similarity between injection sites. **(C)** Hierarchical clustering is used to agglomerate individual projection sites into larger zones. A threshold (dashed line) is chosen to indicate the output division for clustering. Colored lines below indicate the different clusters. **(D)** Similarity matrix sorted by clustering identity shows that clustering indeed finds groups of similar injection sites. Colors from **(D)** indicate clusters. Note additional structure can be found on scales other than the one chosen for clustering **(E)** Clusters are aggregated into zones and the averaged connectivity between these zones is shown as a schematic network representation. Colors indicate cluster identity as in **(C,D)**. Width of lines indicates strength of averaged projection. **(F,G)** Micro-scale descriptions. **(F)** Synaptic distribution on the dendrites is one of the most important determinants for input-output functions of the neuron. Do different branches within a neuron have different synapse density (*upper*) and are synapses on a branch clustered (*lower*)? Significant tropism over subset of dendritic branches and spatial clustering of synapses were demonstrated by ATing and mGRASPing, supporting active dendritic integration. **(G)** Structured synaptic distribution of the reconstructed pyramidal neurons in somatosensory cortex (L5) from thalamus inputs. The density of thalamocortical synapses on dendrites is indicated in color-spectrum. (Panel **G** is a modification of the figure from Rah et al., 2013).

Meso-scale descriptions have been previously offered in terms of an inventory of cell-types (Bohland et al., 2009; Seung and Sümbül, 2014) and by approaches that smooth over fine anatomical details (coarse-graining) arising from microscopic fluctuations, thus allowing for effective mesoscopic descriptions, and the classification of neuronal populations into functional groups (Bohland et al., 2009; Mitra, 2014). Determining the axonal projections from a given set of neurons to target brain areas can assist in defining cell-types (Mitra, 2014). Recently, examples of such efforts have been published (Hunnicutt et al., 2014; Oh et al., 2014; Zingg et al., 2014) and even cell-type-specific connectivity has been reported in a whole brain atlas (Pollak Dorocic et al., 2014). In this work, image alignment, registration, and annotation, connectivity patterns were summarized, as described above, in a large matrix tabulating the projection density between the different injection sites and a list of predefined target areas (Figure 3A). The information found in this series of papers could be used to generate a meso-scale description by a clustering analysis that groups together regions revealed by separate experiments according to the similarity of their input and output projections (Figures 3A–E). Thus, information about anatomical projections can be used to define meso-areas that serve as a mid-level layer of description between the single neuron and the full brain area. This should assist in assigning a more interpretable level of functional roles to neural circuits as well as guiding development of a micro-scale connectome.

Beyond coarse-scale anatomical projection paths, micro-scale descriptions, for instance using AT or mGRASP, can be used to provide a detailed understanding of neuronal signal processing. By identifying the number of synapses in a projection between two groups of neurons, and the specific spatial distribution of individual synapses along the dendritic arbor of the postsynaptic cells, the transfer of signals from one population to another can be studied in far greater detail. Recently, such micro-scale synaptic connectivity mapping with AT and mGRASP provided direct evidence for significant spatial synapse clustering and for a substantial level of structured synaptic distribution over subsets of dendritic branches (Figures 3F,G; Rah et al., 2013; Druckmann et al., 2014).

For many years a number of electrophysiological and theoretical studies have demonstrated the presence of biophysical substrates for local processing on dendritic branches. These studies led to a hypothesis that a structured organization of synapses at the cellular and dendritic levels may also exist to utilize this specialized biophysics (Poirazi et al., 2003). Such an organization may provide advantages for computation. On the biophysical level, spatially clustered, temporally synchronized synaptic inputs tend to be amplified by triggering local dendritic spikes through the opening of voltage-dependent cation channels (such as voltage-dependent sodium channels, voltage-sensitive calcium channels, NMDA receptor channels), which enhance local voltage deflection supra-linearly, thereby increasing the chance of generating action potentials (Magee et al., 1998; London and Häusser, 2005; Magee and Johnston, 2005; Johnston and Narayanan, 2008; Sjöström et al., 2008). However, the technical challenge of determining electrophysiologically whether the distribution of synaptic inputs follows the local structure has made it difficult to test the hypothesis, particularly across neuronal populations.

To extend the hypothesis, one would want to know whether synapses convey *related information (origin, subtype, response specificity etc.)*, in a spatially clustered manner. Multiple functional studies supported this idea that spatially clustered synapses receive related information (Larkum et al., 1999; Poirazi and Mel, 2001; Harnett et al., 2013). Furthermore, it has been shown that dendritic branches have collective response properties to stimuli as shown by the coupling between local dendritic spikes and the somatic voltage change being dependent upon the specific branch rather than individual synapses in the branch (Losonczy and Magee, 2006). The coupling can be modified in a branch-specific manner by plasticity driven by NMDA dependent regulation of local Kv_4.2_ potassium channels (Kim et al., 2007; Losonczy et al., 2008) and dependent upon the excitation history of neighboring dendrites (Remy et al., 2009). Additional studies, performed *in vivo*, that demonstrated distinct functions of dendritic branches support the notion of input clustering (Lavzin et al., 2012; Xu et al., 2012). Using AT and mGRASP, we have directly observed the structured nature of synaptic inputs, by describing both between-branch structure, and within-branch clustering, in terms of micro-scale descriptions in thalamocortical and hippocampal connections (Rah et al., 2013; Druckmann et al., 2014). To achieve micro-scale descriptions of connectivity, functional characteristics of synapses, such as their efficacy and response specificity, need to be pursued with a high-throughput process. We propose that advanced optogenetic and sensory stimulation paradigms paired with activity sensors, such as the recently developed activity history marker, CaMPARI (Fosque et al., 2015), followed by large-scale AT or mGRASP, will provide complete pictures of micro-scale descriptions of input-specific synaptic connectivity. Combining such meso- and micro-scale descriptions will greatly facilitate our understanding of the operations of complex neuronal networks.

## Conclusion and Perspective

Here we reviewed two techniques, AT and mGRASP, which are useful for imaging mammalian synaptic connectivity at multiple scales. Combining the advantage of mGRASP to accurately detect synapses and AT to profile synapses on the molecular level will enable functional assessments that allow building up from network wiregrams to synaptograms, thus revealing the secrets of complex neural circuits. We suggest that, in addition, future endeavors need to focus on linking meso- and micro-scale connectivity maps. The best way to fill the gaps between maps on different scales would involve creating “standardized linkers” such as common 3D reference space. The increasing pace of technology developments for neuroanatomy in the XX1st century make us feel that it is indeed exciting time to be a neuroscientist witnessing our steps towards keys to unlock the mystery of the brain.

## Conflict of Interest Statement

The authors declare that the research was conducted in the absence of any commercial or financial relationships that could be construed as a potential conflict of interest.

## Footnotes

^1^www.MouseConnectome.org and http://connectivity.brain-map.org

## Abbreviations

3D: three-dimensional
AT: array tomography
ATing: AT-assisted circuit mapping
ATLUM: automated tape collecting lathe ultra microtome
C. elegans: Caenorhabditis elegans
CaMPARI: calcium-modulated photoactivatable ratiometric integrator
E-I balance: excitatory-inhibitory balance
EM: electron microscopy
ENABLED: endogenous labeling via exon duplication
FIBSEM: focused ion bean scanning EM
GFP: green fluorescent protein
Kv: voltage-sensitive K+ channel
LM: light microscopy
mGRASP: mammalian GFP-Reconstitution Across Synaptic Partners
mGRASPing: mGRASP-assisted circuit mapping
MRI: magnetic resonance imaging
PALM: Photo-activated localization microscopy
PET: positron emission tomography
PSD: postsynaptic density
SBFSEM: serial block face scanning EM
SIM: Structured illumination microscopy
spGFP: split-GFP fragments
STED microscopy: Stimulated emission depletion microscopy
STORM: stochastic optical reconstruction microscopy
VGLUT: vesicular glutamate transporter.
